# The Development and Validation of a Turkish Insulin Treatment Self-management Scale Child Form (Ages 8-18) and Parent Form

**DOI:** 10.4274/jcrpe.galenos.2019.2019.0026

**Published:** 2019-09-03

**Authors:** Çağrı Çövener Özçelik, Eda Aktaş, Nesrin Şen Celasin, Gülten Karahan Okuroğlu, Şükriye Şahin

**Affiliations:** 1 Marmara University Faculty of Health Sciences, Department of Pediatric Nursing, İstanbul, Turkey; 2 University of Health Sciences Faculty of Nursing, Department of Pediatric Nursing, İstanbul, Turkey; 3 Manisa Celal Bayar University Faculty of Health Sciences, Department of Pediatric Nursing, Manisa, Turkey; 4 Marmara University Faculty of Health Sciences, Department of Fundamentals of Nursing, İstanbul, Turkey; 5 Kocaeli University Faculty of Health Sciences, Department of Nursing, Kocaeli, Turkey

**Keywords:** Insulin treatment, self-management, scale development, type 1 diabetes

## Abstract

**Objective:**

The aim of the study was to develop an Insulin Treatment Self-management Scale; both Child Form and Parent Form for children ages 8-18 with type 1 diabetes.

**Methods:**

Children with type 1 diabetes and their parents participated in the study. Development of a methodologically designed scale was conducted to investigate insulin treatment self-management of children with type 1 diabetes.

**Results:**

A total of 331 children and their parents were recruited. Children and parents completed the data collection tools by themselves. The final scale had two subscales; one was related to cognitive and behavioural expressions regarding insulin treatment (self-efficacy) and the other to emotional aspects of self-maagement of insulin treatment (emotional impacts). The scale was shown to be valid and reliable.

**Conclusion:**

This study was a valid and reliable scale for measuring insulin treatment self-management in children with type 1 diabetes. Thus can be used to assess insulin treatment self-management in children with type 1 diabetes and their parents as well as a tool for effective nursing care.

**What is already known on this topic?**

Personal management of insulin treatment is crucial for the success of diabetes treatment. There is currently no scale which measures insulin treatment self-management in Turkey. The absence of this kind of scale is a risk factor that may negatively affect the success of insulin therapy.

**What this study adds?**

Children are a very vulnerable group in terms of insulin treatment. Measuring insulin treatment self-management with a valid and reliable tool is a guide to health professionals like diabetes nurses, physician in assessing insulin treatment.

## Introduction

Type 1 diabetes is a chronic metabolic disease caused by an autoimmune reaction to pancreatic beta cells which excrete insulin. It is characterized by absolute insulin deficiency. Type 1 diabetes usually begins during childhood or adolescence (1), mostly between the ages of 7 and 15 years. Type 1 diabetes constitutes 5-10% of all diabetic cases (2). Recently, type 1 diabetes incidence has shown a gradual increase. Worldwide, it is estimated that approximately 1,106,500 children between the ages of 0-19 (3) and 96,000 children under the age of 15 (4) live with type 1 diabetes, and that type 1 diabetes develops in 132,600 children every year (3).

The main aim of type 1 diabetes treatment is to ensure the stability of plasma insulin levels (5,6). Currently, there is no universally accepted insulin treatment for type 1 diabetes. Insulin treatment needs to be arranged for each child in an individualised way to provide optimal metabolic control while minimising interference with their psychosocial development (6,7).

Effective diabetes management depends on the harmony of several factors, such as insulin treatment, eating habits, exercise and personal control. Personal management of insulin treatment is crucial for its success. Patients with type 1 diabetes should have certain skills and attitudes, such as being aware of insulin types and treatment options; correct injection techniques; and the importance of giving the right dose at the right time. They should have sufficient information on insulin injection areas, absorption rates, factors affecting insulin absorption and insulin prevention conditions; understanding and overcoming the complications of insulin treatment; and arranging insulin doses according to food intake (8,9).

Teaching insulin management, which is an essential part of diabetes management, to children with type 1 diabetes and their caregivers is a fundamental part of a diabetes treatment plan. This also helps children and their parents to avoid diabetes-related complications such as hypoglycaemia or hyperglycaemia or, if such complications occur, to know how to treat them properly (8,10,11). Providing education and support to the child and parents is crucial for effective management of type 1 diabetes (11).

There do not yet exist, as far as we know, in the literature any tools to measure insulin treatment self-management levels of children with type 1 diabetes. Similarly, no tools are available for parents to evaluate their children’s insulin management levels. Thus, the necessity to evaluate self-management skills regarding insulin treatment has emerged for both children and their parents. The present study was conducted in order to develop the Insulin Treatment Self-management Scale: Child Form and Parent Form for children of ages 8-18 with type 1 diabetes.

## Methods

### Participants

It has been suggested that, when developing a new scale or questionaire, the sample size should be 5-10 times greater than the total number of items in the scale (12,13,14). Concordantly, because the scale developed for this study included 50 items, the planned sample size was 250-500 participants. The study was thus conducted on 331 children with type 1 diabetes and their parents, as volunteer participants. The inclusion criteria for the children participants were: being followed-up on an outpatient basis; being between 8-18 years of age; having been diagnosed for a minimum of one year; using insulin; not having any other illnesses apart from diabetes; and not being hospitalised during the data collection phase. The inclusion criteria for their parents was not being under psychiatric treatment.

### Procedure

**Formation of an Item Pool:** The item pool was primarily formed during the development of the Insulin Treatment Self-management Scale: Child Form and Parent Form. For both forms, 44 items were generated by the researchers in accordance with the literature (1,2,3,4,5,7,8,11). Items on the parent form were designed for them to evaluate their children. For instance, the item “I apply my injection as it was taught” on the child form was modified to “My child applies his/her injection as it was taught” for the parent form. Each of the items was prepared using a 5-point Likert-type scale ranging from 1 to 5, where 1 denotes “strongly disagree” and 5 “strongly agree”. Scales were filled by scoring them one by one.

**Content Validity:** One of the logical methods to test the content validity of a study is to obtain expert opinions (15). The opinions of 14 experts were requested to assess the comprehensibility of the scale. This expert team consisted of clinicians and academic nurses focusing on diabetes. Furthermore, the content validity index (CVI) was utilised in order to prove both cultural and language equivalence and content validity in numeric values as well as a broad assessment of expert opinions (13). Experts assessed each of the items according to the Davis method (1992) (16), scoring them between 1 and 4, where 1=not appropriate, 2=the item should be reviewed, 3=appropriate, but little changes needed and 4=definitely appropriate. Following the assessment of scores by each of the experts, the items that received a 1 or 2 assessment were removed from the scale and redesigned. The CVI score is defined as 0.80 when 80% of the items score between 3 and 4. Having a score of 0.80 or above suggests appropriate content validity for the study (13). For this questionnaire, none of the items received a score of 1 or 2. Minor changes were made to the items that received a score of 3 in line with the experts’ opinions. In addition, six more items recommended by experts were added to the scale, and their content validity was again tested as described.

**Face Validity:** Regarding scale development studies, the literature suggests that the outline of the scale should be tested with a similar sample group (17,18). Following the language and content validity, 15 children and their parents were given the pre-application form by researchers in order to ensure necessary arrangements like complicated sentences or grammer mistakes in data collection tools to assess the face validity of the scale. Finally, the implementation phase was begun using the 50-item form.

### Data Analysis

The data were analysed by Number Cruncher Statistical System 2007 (Kaysville, Utah, USA). Expert views were evaluated by CVI. The construct validity was assessed by a factor analysis. The reliability analysis of the scale was analysed as follows. Internal consistency was assessed using Cronbach’s alpha coefficient and item total correlation, parallel form reliability was checked by Spearman’s correlation analysis and split-half reliability was calculated. Socio-demographic data was analysed by descriptive statistical analysis [mean, standard deviation (SD) and percentage].

### Data Collection

The study was carried out in İzmir and İstanbul, Turkey, in hospitals with pediatric diabetes centres, between June 2016 and December 2017. These hospitals were selected because they have high populations of paediatric diabetes. Children and parents filled out the data collection tools by themselves. Duration of data collection was recorded as minutes by researchers for each of participants separately.

### Ethical Considerations

Ethical permission was obtained from the Medical Faculty Clinical Researches Ethic Committee of Marmara University (IRB no: 15.07.2016 09.2016.432). In addition, written permission was received from the participating hospitals. Participants were informed about the study, and written consent was obtained from them.

## Results

### Patients with Type 1 Diabetes

A total of 171 (51.7%) of the participating children were girls, and 160 (48.3%) were boys, making a total of 331 patients. For the whole group the mean±SD chronological age was 14.25±2.84 (range: 7-18) and mean±SD age at diagnosis was 6.08±4.00 (range: 1-17) years. The mean±SD HbA1c value of the subjects was 8.92±2.14. Of the parents who participated, 81.0% (n=268) were mothers, 17.5% (n=58) were fathers and 1.5% (n=5) were other guardians. Data collection tools were filled out by participants in minimum 15 minutes and maximum 20 minutes.

### Content Validity

The adjustments were proved for expert views on Insulin Treatment Self-management Scale Child Form and Parent Form according to Kendall’s W adjustment analysis realised to ensure content validity (Kendall’s W^a^_Child Form_=0.109, df=41, p=0.170; Kendall’s W^a^P_arent Form_=0.009, df=43, p=0.420). The CVI, analysed via the opinions of experts according to the Davis method (1992) (16), was 0.93 for the child form and 0.94 for the parent form. Fifteen children and their parents were given the pre-application form by researchers and Cronbach’s alpha coefficient for child form was 0.87 and parent form was 0.88.

### Construct Validity

An exploratory factor analysis (EFA) was conducted in order to identify the structure of the scale. In addition, the Kaiser-Meyer-Olkin (KMO) test and Bartlett’s test of sphericity were applied in order to determine the appropriateness of the data to the factor analysis. The KMO value was 0.889 for the child form and 0.901 for the parent form. The sphericity was statistically significant for both of the forms (child form: χ^2^=4417.66, p<0.001; parent form: χ^2^=4511.27, p<0.001).

**Figure 1:**
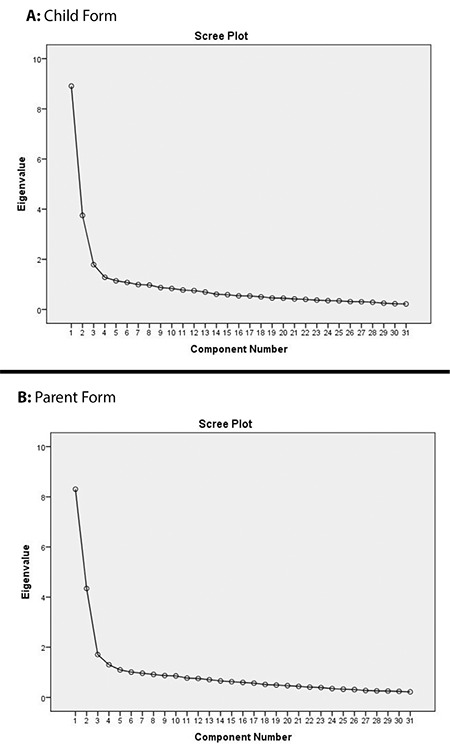
A, B). The scree plots present the factorial structure of the scale

For the EFA, the varimax vertical rotation technique was applied. As a result of the analysis, 10 items with an item load below 0.30 and nine items with loads from multiple factors were removed from the child and parent forms. The variant analysis showed that both of the forms had a two-factor structure. The two-factor structure explained 40.79% of the total variance for the child form and 40.82% for the parent form. For the child form, the first factor explained 26.78% of the variance, and the second one explained 14.00%. For the parent form, the first factor explained 28.73% of the variance, and the second one explained 12.09%. Scree plots present the factorial structure of the scale (Figures 1A, 1B).

**Factor 1**: Items 1-10, 13-17, 21, 22, 24 and 27-31 were gathered under this factor. These items include cognitive and behavioural expressions regarding insulin treatment. Thus, the factor was named self-efficacy.

**Factor 2:** Items 11, 12, 18-20, 23, 25 and 26 were gathered under this factor. These items include negative emotional expressions. Thus, the factor was named emotional impacts.

The item loads ranged from 0.42 to 0.83 for the child form and from 0.40 to 0.80 for the parent form. The findings obtained from the EFA are presented in Table 1.

### Reliability

Item total correlation, inner consistency reliability, split-half test and parallel test techniques were utilised in order to test the reliability of the scale. The item total correlation for the Insulin Treatment Self-management Scale ranged from 0.21 to 0.58 for the child form and from 0.25 to 0.64 for the parent form (Table 1). In order to determine the inner consistency reliability of the scale, Cronbach’s alpha was used. To determine two halves reliability, Spearman-Brown and Guttman split-half coefficients were calculated. These values are presented in Table 2. The correlation between the child and parent forms was examined for parallel test reliability, and the results are presented in Table 3.

### Scoring the Insulin Self-management Scale

The 5-point Likert-type scale includes 31 items and two sub-groups. The first factor consists of 23 items. The minimum score for this factor was 23 and the maximum was 115. Higher scores imply a higher level of self-efficacy. The second factor consists of eight items with a minimum score of eight and a maximum score of 40. The items for this factor have a reverse scoring. After reverse scoring, higher scores indicate the respondent’s positive feelings towards insulin management.

The overall score of the scale ranged from 31 (minimum) to 155 (maximum). Higher scores indicated a higher level of self-management regarding insulin treatment.

## Discussion

Development of the scale began by searching for similar studies in the literature. However, no specific scales for measuring the self-management skills of children with type 1 diabetes were found in either our country or in others. This scale is important for identifying insulin treatment self-management skills as well as nursing care and self-management needs and to attain a desired level by the diabetes nurses. It is also important for developing individualised education programmes.

This methodologically designed scale was created to identify insulin treatment self-management for children with type 1 diabetes. A newly-developed scale should meet two important criteria: validity and reliability. Validity refers to how well a scientific test or a scale actually measures what it sets out to or how well it reflects the reality it claims to represent. Thus, if a scale correctly measures what it sets out to without interfering with other factors, then that scale can be accepted as valid (16). A valid scale should be reliable. Reliability is defined as the consistency between participants’ responses to the scale’s items (15). Content and construct validity were utilised in our study to test the reliability.

### Content Validity

Content validity is the indicator of how sufficiently the items qualitatively and quantitatively measure the intended behaviour (15,19). According to the results of Kendall’s W adjustment analysis, no significant differences were detected between the experts’ opinions of the scale. Such a result shows that the items were understood similarly by the experts. Thus, the scale to measure insulin self-management skills was comprehensible.

### Construct Validity

Construct validity refers to the degree to which a test measures a discrete concept in terms of desired behaviours. One of the techniques to test construct validity is factor analysis (15). The EFA is a technique to determine the number of sub-groups of items in a scale as well as the relation between them (17,20). The EFA was used to test the construct validity of the scale. However, prior to the EFA, the KMO test and Bartlett’s test of sphericity were utilised in order to determine whether the number of samples was sufficient and if there was a desired level of relation between the variables. The KMO test is an index comparing observed correlation coefficients with partial correlation coefficients. The KMO values range between 0 and 1, and a value >0.80 is expected for a successful factor analysis. In Bartlett’s test of sphericity, having a p<0.05 indicates an appropriate level of relation among variables for a factor analysis (21). It has been reported that a KMO value >0.50 is enough to realize a factor analysis (13,15). In the present study, the KMO value for the Insulin Treatment Self-management Scale was 0.88 for the child form and 0.90 for the parent form, indicating its suitability for factor analysis. Furthermore, for Bartlett’s test of sphericity, the p value was significant for both the child (p<0.001) and parent (p<0.001) forms, indicating that the correlation matrix for the items in the scale is suitable for the factor analysis.

The eigenvalue of items in the factor analysis should be at least 1.00, and the item factor load value should be at least 0.30 with a difference of at least 0.20 between items to have enough factor load between two different factors (20). The result of the factor analysis was 2.00. The scree plots present the factorial structure of the scale (Figures 1A, 1B). According to the scree plot, the distance between two points is accepted one factor and following the second factor the distance between two points was both little and similar (20) so that the scale was accepted as possessing two-factors. It is not recommended to have a factor load below 0.30 (21). Regarding the factor load, 0.71 and above is accepted as perfect, 0.63 is very good, 0.55 is good, 0.45 is acceptable and 0.32 is weak (22). In our study, the factor loads were high (Table 1), which confirmed the structure of the scale was appropriate.

### Reliability

Reliability is the extent to which a scale measures what it sets out to measure (15,18,23,24). Reliability emphasises consistency (a factor affecting the validity) that does not change with time. Although a valid test is always reliable, a reliable test is not always valid (15,18).

The reliability of the Insulin Treatment Self-management Scale was tested through internal consistency, the split-half test and item total score correlation techniques. Internal consistency refers to the extent to which characteristics and mean behaviours are similar to each other (15). One of the most common methods to test reliability is Cronbach’s alpha. A Cronbach’s alpha coefficient of 0.00<α<0.40 shows that the scale is not reliable, 0.40<α<0.60 indicates low reliability, 0.60<α<0.80 indicates reliability and 0.80<α<1.00 shows high reliability (25,26). For our scale, the Cronbach’s alpha was 0.86 for the child form and 0.88 for the parent form, indicating high reliability.

Item total correlation explains the relation between the scores obtained from each of the test items and the total score. A higher item total score correlation indicates a higher level of internal consistency and similar sampling behaviours. It has been suggested that items with a score of 0.30 and greater differentiate the participants quite well and should be kept, 0.20-0.30 might be removed and below 0.20 should be removed from the scale (15). For Items 6, 11 and 20 on the child form and 11, 20, 26 and 28 on the parent form, the total score correlations were between 0.20 and 0.30. However, the factor loads for these items were between 0.40 and 0.64, so they were retained in the scale.

One of the most common ways to test the reliability of a scale is to use the split-half test technique. Split-half test reliability refers to the correlation coefficient calculated for the overall scale in that test items’ being separated into two halves and by using the correlations of these two halves with Spearman-Brown formulas and Guttman split-half formulas (15). Having a reliability coefficient of 0.70 or higher indicates a reliable measurement for the scale (17,20). In our study, the Spearman-Brown split-half test correlation was 0.70 for the child form and 0.75 for the parent form. The Guttman split-half coefficient was 0.70 for the child form and 0.75 for the parent form (Table 2). These reliability coefficients show that both forms have reliable measurements.

In order to ensure the reliability of the parallel forms, the correlation between the child form and parent form was examined. The correlation coefficients used were the Pearson correlation coefficient and Spearman’s correlation coefficient (27). Both the Pearson correlation coefficient and Spearman’s correlation coefficient (r) measure the strength of the linear association between variables. The value of a correlation ranges between -1 and +1. Negative values indicate a negative linear relation and positive values indicate a positive linear relation. Both the Pearson correlation coefficient and Spearman’s correlation coefficient are interpreted as follows: 0.00=no correlation, 0.01-0.29=lower-level correlation, 0.30-0.70=mid-level correlation, 0.71-0.99=high-level correlation and 1.00=perfect correlation (28). For this measurement, a high-level positive correlation was found between the two scales (r=0.71, p<0.001) (Table 3).

### Study Limitations

In this study, we were unable to determine the test-retest reliability due to time limitations.

## Conclusion

There is strong evidence that the psychometric characteristics of the scale are valid and reliable. In this study, a valid and reliable scale was developed in order to measure insulin treatment self-management of children with type 1 diabetes and their parents In addition, since there is no similar scale in the literature, it could be used in future studies on this issue.

## Figures and Tables

**Table 1:**
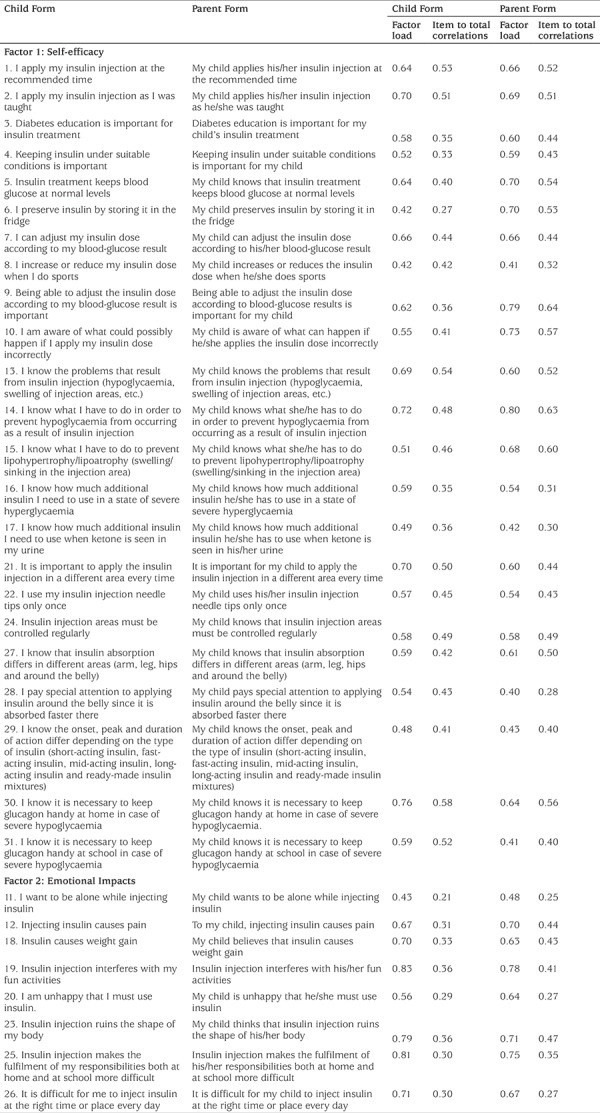
Characteristics of subscales of the Insulin Treatment Self-management Scale: Child Form and Parent Form (n=331)

**Table 2:**
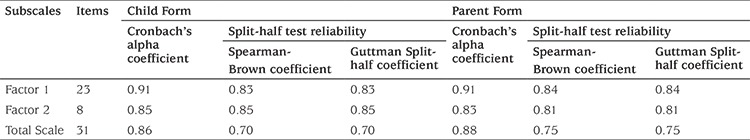
Cronbach’s alpha and Split-Half Test Reliability Results for the Insulin Treatment Self-Management Scale: Child Form and Parent Form

**Table 3:**
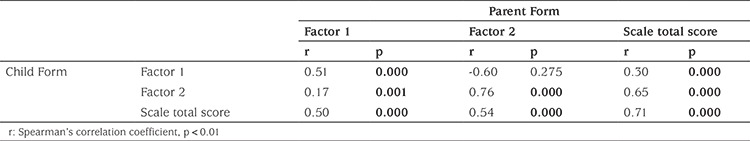
Intercorrelation (Parallel Form reliability) between the Parent and Child Forms for the Insulin Treatment Self-management Scale (n=331)
